# A research agenda for non-communicable disease prevention and control in India

**DOI:** 10.1186/s12961-020-00639-0

**Published:** 2020-10-30

**Authors:** Ishu Kataria, Mariam Siddiqui, Theresa Gillespie, Michael Goodman, Preet K. Dhillon, Carla Bann, Linda Squiers

**Affiliations:** 1 Center for Global Noncommunicable Diseases, RTI International, New Delhi, India; 2 Winship Cancer Institute, Department of Surgery, Emory University, Atlanta, GA United States of America; 3 Rollins School of Public Health, Department of Epidemiology, Emory University, Atlanta, GA United States of America; 4 Center for Chronic Disease Control and Public Health Foundation of India, New Delhi, India; 5 RTI International, Durham, NC United States of America

**Keywords:** Non-communicable diseases, India, Delphi, Snow Card, Prevention, Control, Primary Care, Universal Health Coverage, Health System Strengthening, Integrated Care

## Abstract

**Background:**

Non-communicable diseases contribute to 62% of total deaths in India; of concern are the preventable premature deaths, which account for a staggering 48% of mortality. The objective of this study was to establish a consensus research agenda for non-communicable disease prevention and control that has the potential to impact polices, programmes and healthcare delivery in India.

**Methods:**

To develop a non-communicable disease research agenda, we engaged our community collaborative board and scientific advisory group in a three-step process using two web-based surveys and one in-person meeting. First, the Delphi methodology was used to generate topics. Second, these ideas were deliberated upon during the in-person meeting, leading to the prioritisation of 23 research questions, which were subjected to Strength, Weakness, Opportunities and Threat analysis by the stakeholders using the Snow Card methodology with the scientific advisory group and community collaborative board. This step resulted in the identification of 15 low effort, high impact priority research questions for various health outcomes across research disciplines based on discussion with the larger group to reach consensus. Finally, the second web-based survey resulted in the identification of 15 key priority research questions by all stakeholders as being the most important using a linear mixed effect regression model.

**Results:**

The final set of 15 priority research questions focused on interventions at the individual, community, systems and policy levels. Research questions focused on identifying interventions that strengthen healthcare systems and healthcare delivery, including models of care and improved access to non-communicable disease screening, diagnosis and treatment, determining the impact of government policies, assessing the effectiveness of prevention programmes (e.g. tobacco, environmental improvements), and testing research tools and resources to monitor non-communicable diseases at the population level.

**Conclusion:**

To produce the evidence base for selecting and implementing non-communicable disease programmes and policies in India, investments are needed. These investments should be guided by a national research agenda for the prevention and control of non-communicable diseases in India. Our findings could form the backbone of a national research agenda for non-communicable diseases in India that could be refined and then adopted by government agencies, the private sector, non-governmental and community-based organisations.

## Background

Non-communicable diseases (NCDs) account for approximately 41 million deaths each year; 15 million of these deaths are considered premature (before the age of 70 years) [1] and over 85% occur in low- and middle-income countries [2]. In India, NCDs contribute to 62% of total deaths; of particular concern are preventable premature deaths, which account for a staggering 48% of the overall mortality [3]. The leading causes of NCD mortality in 2016 were cardiovascular diseases, chronic respiratory diseases, cancers, diabetes, and urogenital, blood and endocrine diseases [4]. Findings from the India state-level Disease Burden Initiative reveal dramatic increases in disability-adjusted life year rates between 1990 and 2016 for two specific NCDs – diabetes and ischemic heart disease. The high prevalence of major risk factors, such as tobacco use, harmful consumption of alcohol, poor diet and physical inactivity, are contributing to the rising burden of NCDs [5]. If the four major NCDs (diabetes, cardiovascular disease, chronic respiratory disease and cancer) are left unaddressed, they could cost India approximately US$ 3.55 trillion in economic losses by 2030 [6].

In order to effectively address the growing burden of NCDs, there is a need for adequate country level data, which are currently lacking in India [7]. Comprehensive monitoring of epidemiological trends for NCD risk factors will be crucial to accurately estimate disease burden and inform policy and programme implementation and evaluation [8]. The Government of India (GoI) has committed to meeting the NCD-related Sustainable Development Goal target, which states that “*by 2030, reduce by one third premature mortality from non-communicable diseases through prevention and treatment and promote mental health and well-being*” [9]. To achieve this target, GoI has launched the National Programme for Prevention and Control of Cancer, Diabetes, Cardiovascular Diseases and Stroke (NPCDCS) with a focus on strengthening infrastructure, human resources, health promotion, early diagnosis, treatment and referral [10]. In July 2017, India presented its Voluntary National Review on the Implementation of Sustainable Development Goals at the High-Level Political Forum held at United Nations Headquarters in New York [11]. So far, the GoI has established various NCD cells, which are responsible for managing the NPCDCS programme and are tasked to implement and oversee NPCDCS’ programme activities at state, district, community health centre and sub-centre levels. The activities addressed by the NCD cells include health promotion, early diagnosis, treatment and referral, building strong monitoring and evaluation systems through the public health infrastructure, and disease-specific care centres at district and state level across the country [11].

To address the NCD challenge, India will need to use interventions shown by research to reduce the risk, prevalence and incidence of NCDs. However, the number of well-studied and tested NCD-related interventions, policies, infrastructure investments and changes to health systems in India is relatively small [12]. Concerted efforts and collaborative research will be required to determine which interventions are most effective for different populations throughout India. However, the research infrastructure to produce these evidence-based interventions is lacking.

To help address this gap in research infrastructure, the National Institutes of Health (NIH) provided Emory University with a 2-year planning grant to develop a Regional Center of Research Excellence (RCRE) in NCDs in India (Planning grant for Regional Centers of Research Excellence in Non-communicable Diseases). Activities under this grant were conducted with support from the Public Health Foundation of India and RTI International and focused on the northern and southern regions of India. A key activity of the RCRE was to develop a comprehensive research agenda for NCD prevention and control in India. At the time the RCRE was formed, an agenda to establish priorities for research to inform NCD prevention and control efforts and guide funding in India did not exist. The purpose of this manuscript is to describe the methods used to develop a consensus-based set of research priorities and share the results. These results can be used as the foundation for the development of a research agenda for NCD prevention and control in India.

## Methods

To develop an NCD research agenda for India, we engaged a broad range of stakeholders in the fields of NCD policy, public health, research, clinical care and leadership to ensure diverse perspectives were represented during consensus building. We included individuals who served on the community collaborative board (CCB) and the scientific advisory group (SAG) who were a part of the RCRE planning grant. The SAG included scientists with established expertise in NCD research and various aspects of infrastructure development, research, and research enhancement and policy relevant for the region whereas the CCB included national- and state-level stakeholders promoting attention to community interests, strengthening relationships between investigators and stakeholders involved in NCD prevention and treatment. The members covered various disciplines and had a north–south distribution amongst themselves (Table 1).

**Table 1:** Stakeholder profile for research agenda development

Organisation	Organisation description	Type	
		CCB	SAG
1. Indian Council of Medical Research	Government		✓
2. Indian Institute of Public Health	Academic and research		✓
3. American Cancer Society	NGO/non-profit		✓
4. Medanta – The Medicity	Healthcare (private)		✓
5. Cancer Foundation of India	NGO/non-profit	✓	
6. Cancer Institute (WIA)	Research	✓	
7. CanSupport	NGO/non-profit	✓	
8. Coimbatore Cancer Foundation	NGO/non-profit	✓	
9. IKP Centre for Technology in Public Health	NGO/non-profit	✓	
10. Medic Mobile	NGO/non-profit	✓	
11. Ministry of Health and Family Welfare	Governmental	✓	
12. PSG Institute of Medical Sciences Research	Healthcare (private), academic and research	✓	
13. Tamil Nadu Health Systems Project	Governmental	✓	
14. Viamo	Social enterprise	✓	
15. Indian Cancer Society	NGO/non-profit	✓	
16. International Agency for Research on Cancer	NGO/non-profit		✓
17. All India Institute of Medical Sciences	Healthcare (public)		✓
18. Public Health Foundation of India	Research		✓
19. RTI International	NGO/non-profit		✓

*CCB* Community Collaborative Board, *NGO* Non-governmental Organisation, *SAG* Scientific Advisory Group

We implemented a three-step process that incorporated both qualitative and quantitative research methods to identify, generate and prioritise research questions that, if answered, had the potential to reduce the incidence of NCDs in India. Table 2 presents an overview of each step, its purpose, the data collection method used and the final product developed.

**Table 2:** Steps to gathering inputs for research agenda development

Step	Purpose	Method	Product
Step 1: Delphi	• Collect and analyse participants’ ideas for critical research that should be conducted in different disciplines and for different NCDs • Group ideas into broad themes	Online survey	List of priority research questions
Step 2: Snow Card	• Share results from Step 1 and present to CCB and SAG • Group conducts a SWOT analysis of the ideas generated in Step 1 Participants are divided into groups, with both CCB and SAG members included and asked to categorise ideas based on level of effort (low vs. high) and impact generated (low vs. high) considering their organisational capacity	In-person	List of high impact, low effort research questions
Step 3: Follow-up	Generate a final list of 15 prioritised ideas based on those identified as low effort, high impact areas during in-person meeting	Online survey	List of 15 research questions that could be pursued further

*CCB* Community Collaborative Board, *NCDs* Non-communicable Diseases, *SAG* Scientific Advisory Group, *SWOT* Strengths, Weaknesses, Opportunities and Threats

### Step 1. Idea generation

To generate ideas about research that should be conducted to reduce the incidence of NCDs in India, we used the Delphi method, which is a consensus building process that gathers diverse perspectives and organisational agendas from a variety of different experts or stakeholders. Using an iterative approach, stakeholders rate and rank the different ideas that are shared by experts or stakeholders [13]. To collect stakeholders’ perspectives about what they saw as important research questions that needed to be answered to reduce the incidence of major NCDs in India, we conducted an online survey with all stakeholders from both SAG and CCB (*n* = 30). We asked participants to list at least two priority research questions for each of the following health issues: cardiovascular disease, cancer, diabetes, chronic respiratory diseases and mental health. We used these main category descriptors for NCDs to be in line with the new WHO 5 × 5 framework for NCDs [14]. Next, we asked participants to identify research questions or issues for each of the following research disciplines: epidemiology, clinical research, biostatistics, health systems strengthening, health economics, health promotion, implementation science and health policy. For each research question, we asked participants to share the reason they prioritised the question. A total of 19 participants responded (63.3%), helping generate 165 research questions. After accounting for clustering and duplication, we had 56 unique research questions.

### Step 2. Shared prioritisation

To obtain feedback on the 56 unique research questions, we held an in-person meeting where these 19 participants had the opportunity to review and deliberate about the research questions identified in Step 1. The 56 research questions were listed on four large sheets of paper and taped around the room. Each participant was given a set of 10 stickers and asked to place them next to the research question(s) that they felt were most important for the prevention and control of NCDs in India. Participants did this exercise individually, without discussing their selections with other participants. Each participant could either place all 10 stickers on one of the 56 research questions or distribute them among the many research questions based on their relative importance. This resulted in the identification of 23 research questions that were assumed to be most important as they received maximum votes. We then asked participants to perform a small group SWOT (Strength, Weakness, Opportunity and Threat) analysis, which was done using Snow Card methodology – a nominal group technique used to identify and analyse the strengths, weaknesses, opportunities, and challenges in a strategic planning process. Through this method, the diverse ideas expressed in a group of participants can be integrated to represent agreement on a particular issue [15]. We organised participants into five groups. We assigned each group four or five priority research questions. For every priority research question, each individual in the group listed the Strength(s) and Weakness(s) of the research question, as well as the Opportunities for success that the research question could bring and the Threats, or opposition or challenges in implementing the research question, and what their own organisation could contribute toward addressing the research question. This individual exercise was followed by a small group discussion, in which members discussed the collective assessments of the research questions’ priorities assigned to them until they reached consensus. A volunteer from each group was asked to report the results of the SWOT analysis back to the larger group. The final step was to categorise prioritised research questions within the larger group based on level of effort (low versus high) and potential to impact (low versus high). This categorisation exercise resulted in a set of 15 priority research questions that were deemed by the group as low effort, high impact.

### Step 3: Final prioritisation

All the stakeholders who were a part of the in-person meeting were invited to complete a second online survey to rank the final set of 15 research questions. They ranked the research questions in order of importance (1 = most important to 15 = least important). We computed the mean of these rankings across stakeholders for each of the research questions. Because the same individuals ranked each research question, we treated the values as repeated measurements and analysed them using a linear mixed effect regression model. In this model, the outcome was ranking (i.e. 1 to 15), research question was a fixed effect predictor variable and person was a random effect. Within this model, contrast statements were used to compute pairwise comparisons between research questions. A significant difference between a pair of research questions would indicate that stakeholders ranked one research question significantly higher (or lower) than the other.

## Results

The least squares mean of ranking for each research question and the main issue the research question addresses are presented in Table 3. The order in which the questions are listed in the table is the final ranking of the priorities. The lowest mean ranking, which means the topic that was ranked as most important, was for ‘Development of interventions to empower primary physicians and health workers in early diagnosis of NCDs’. The topic that had the lowest mean ranking was understanding the ‘Impact of enabling environment (e.g. green spaces for promotion of physical activity) to prevent and control NCDs’. Priorities focused on identifying interventions that strengthen healthcare systems (Q2) and healthcare delivery, including models of care (Q3) and improved access to NCD screening, diagnosis and treatment (Q1, Q5, Q6, Q8). Research that examines how government policies (Q7) and mandates, such as adding HPV vaccination to the National Immunization Programme (Q12), affect the incidence and prevalence of NCDs were also prioritised by stakeholders. Prevention programmes, such as tobacco prevention (Q9), environmental improvements [16] and the role of Ayurveda, Yoga & Naturopathy, Unani, Siddha and Homeopathy (Q14), were also proposed as priorities. Finally, research tools (Q4) and resources (Q11) to monitor NCDs at the population level were deemed important as was understanding factors that improve the quality of life of people with NCDs.

**Table 3:** Research questions in priority order: results of linear mixed effects regression model

Questions	Least squares mean	Priorities	
Q1. Which interventions are effective in empowering primary physicians and healthcare workers to prioritise the early diagnosis of NCDs among their patients?	5.11	**Top 5 priorities**	**Top 10 priorities**
Q2. What type of health systems strengthening programmes are effective in increasing the control of NCDs?	6.16		
Q3. Are integrated care models to address multi-morbidity (coexisting multiple chronic conditions) such as tuberculosis, chronic obstructive pulmonary disorder, diabetes and cardiovascular disease feasible in primary care?	6.26		
Q4. Which tools can effectively monitor population trends for risk factors and for NCD morbidity and mortality in India?	6.42		
Q5. Which strategies are most effective in mobilising different subgroups of the population (e.g. age, religion, income) to participate in NCD screening?	6.58		
Q6. To what degree are treatment options for each NCD available, accessible and affordable in India? Where are the gaps?	6.84		
Q7. To what degree do government policies support the prevention and control of NCDs in India?	7.00		
Q8. What low cost, indigenous, point-of-care tools are available or need to be developed in diagnosing and treating cardiovascular disease and diabetes?	7.79		
Q9. Which interventions are effective in preventing tobacco use among adolescents (10–19 years old)?	7.95		
Q10. Are current options for NCD screening and treatment in private care facilities affordable and effective?	8.68		
Q11. What is the feasibility of establishing a national NCD registry to create a unified framework for NCD surveillance?	8.95		
Q12. Is it feasible to add HPV vaccination to the National Immunization Programme?	8.95		
Q13. What factors affect the quality of life in patients living with a single NCD (e.g. cancer, diabetes) or with multiple NCDs?	10.68		
Q14. What role can the system of Ayurveda, Yoga and Naturopathy, Unani, Siddha and Homeopathy have in NCD prevention?	11.00		
Q15. What impact do environmental improvements to promote healthy lifestyles have on the prevention and control of NCDs?	11.63		

*NCD* Non-communicable Disease

## Discussion

Interventions that have been tested regionally and locally are needed to reduce the incidence, prevalence, morbidity and mortality of NCDs. By creating the NPCDCS Programme, the GoI has signalled that reducing NCDs is a priority and research to create the evidence-base for programmatic efforts needs to be funded. An NCD research agenda for India is needed to guide funding and establish research priorities. Our findings represent research questions that (1) have the potential to have a high impact, (2) are relatively feasible to answer in terms of level of effort, and (3) have been vetted by knowledgeable stakeholders representing a variety of different disciplines and fields involved in NCD prevention and control in India. The research questions included identifying and testing interventions at the individual, community, systems, and policy levels and can contribute to a national research agenda for NCD prevention and control in India.

Having an established and promoted research agenda to guide funding and researchers would allow stakeholders, such as GoI, academia, healthcare delivery institutions, healthcare providers and communities, to develop collaborative research that answers the most important research questions that have the potential to reduce the burden of NCDs in India.

It is important that we acknowledge the limitations of our approach and how these may have affected our findings. First, the study participants were recruited as either members of the SAG and CCB developed for the NIH-funded RCRE for NCDs, which focused on two regions of India – New Delhi in the north and Chennai in the south. When we asked participants to share their opinions about the most important research questions that needed to be answered, we did ask participants to focus on the country of India in its entirety. However, because participants worked in predominantly in these two regions, their opinions may have been shaped by their current work and personal experiences in these regions. Second, the wording and framing of the questions we asked participants in all three steps of the prioritisation process likely influenced their responses. For example, when we original solicited research questions participants believed to be important, we asked about the specific health issues, namely cardiovascular disease, cancer, diabetes, chronic respiratory diseases and mental health. We did not include other categories of NCDs (e.g. accidents and injuries) or call out stroke separately from cardiovascular disease as has been done by others [16]. Our word choice (e.g. using the term chronic respiratory diseases versus lung disease) may have affected the research questions generated by participants. Third, we asked participants to prioritise research questions that would have a high impact and low level of effort so that we ended up with a list of priorities that was feasible to address. As a result, our results do not represent research that could have a high impact and high level of effort. Even with these limitations, these research questions can serve as the beginnings of or as the impetus for a formal research agenda that can support the development and implementation of cost-effective strategies to prevent and control NCDs in India, thereby achieving our NCD targets.

## Conclusion

Investments in research are needed to produce the evidence base for selecting and implementing NCD programmes and policies in India. These efforts should be guided by a national research agenda for the prevention and control of NCDs in India. Our findings could form the backbone of a national research agenda for NCDs in India that could be refined and then adopted by government and private, non-governmental and other community-based organisations.

## Data Availability

All data generated or analysed during this study are included in this published article.

## Abbreviations

CCB: Community Collaborative Board
GoI: Government of India
NCDs: Non-communicable Diseases
NPCDCS: National Programme for Prevention and Control of Cancer, Diabetes, Cardiovascular Disease and Stroke
RCRE: Regional Center for Research Excellence
SAG: Scientific Advisory Group
SWOT: Strength, Weakness, Opportunity, and Threat

## References

[1] World Health Organization. Global Action Plan for the prevention and control of noncommunicable diseases 2013–2020. 2012. https://apps.who.int/iris/bitstream/handle/10665/94384/9789241506236_eng.pdf;jsessionid=52E1B3C677527E0D4DB828DDB046B5A3?sequence=1. Accessed 21 Jan 2019.

[2] World Health Organization. Noncommunicable diseases factsheet. 2019. http://www.who.int/news-room/fact-sheets/detail/noncommunicable-diseases. Accessed 19 Jan 2019.

[3] India State-Level Disease Burden Initiative Collaborators (2017). Nations within a nation: variations in epidemiological transition across the states of India, 1990-2016 in the Global Burden of Disease Study. Lancet.

[4] Indian Council of Medical Research, Public Health Foundation of India, Institute for Health Metrics and Evaluation. India: Health of the Nation’s States—The India State-Level Disease Burden Initiative. New Delhi: ICMR, PHFI, and IHME; 2017. https://phfi.org/wp-content/uploads/2018/05/2017-India-State-Level-Disease-Burden-Initiative-Full-Report.pdf. Accessed 20 Jan 2019.

[5] Sinha R, Pati S (2017). Addressing the escalating burden of chronic diseases in India: need for strengthening primary care. J Family Med Prim Care.

[6] Bloom DE, Cafiero-Fonseca ET, Candeias V, Adashi E, Bloom L, Gurfein L, et al. Economics of Non-Communicable Diseases in India: The Costs and Returns on Investment of Interventions to Promote Healthy Living and Prevent, Treat, and Manage NCDs. Boston: World Economic Forum, Harvard School of Public Health; 2014. http://www3.weforum.org/docs/WEF_EconomicNonCommunicableDiseasesIndia_Report_2014.pdf. Accessed 21 Jan 2019.

[7] Nethan S, Sinha D, Mehrotra R (2017). Non communicable disease risk factors and their trends in India. Asian Pac J Cancer Prev.

[8] Raban MZ, Dandona R, Dandona L (2012). Availability of data for monitoring noncommunicable disease risk factors in India. Bull World Health Organ.

[9] United Nations. SDG3: Good Health and Wellbeing. https://in.one.un.org/page/sustainable-development-goals/sdg-3-2/#:~:text=By%202030%2C%20end%20the%20epidemics,mental%20health%20and%20well%2Dbeing. Accessed 21 Jan 2019.

[10] Ministry of Health & Family Welfare. National Programme for Prevention and Control of Cancer, Diabetes, Cardiovascular Diseases and Stroke (NPCDCS). New Delhi: MoHFW; 2013. https://mohfw.gov.in/sites/default/files/Operational%20Guidelines%20of%20NPCDCS%20%28Revised%20-%202013-17%29_1.pdf. Accessed 19 Feb 2019.

[11] Ministry of Health & Family Welfare. Voluntary National Review Report on the Implementation of Sustainable Development Goals. New Delhi: MoHFW; 2017. https://sustainabledevelopment.un.org/content/documents/15836India.pdf. Accessed 19 Feb 2019.

[12] Arokiasamy P (2018). India's escalating burden of non-communicable diseases. Lancet Glob Health.

[13] Gilmore GD. Multi-step surveys: the Delphi technique. In: Gilmore G, Campbell D, editors. Needs and Capacity Assessment Strategies for Health Education and Health Promotion. Sudbury: Jones & Bartlett Learning; 2004.

[14] UN General Assembly (73rd session: 2018–2019). Political declaration of the 3rd High-Level Meeting of the General Assembly on the Prevention and Control of Non-Communicable Diseases: resolution / adopted by the General Assembly. New York: UN; 2018. https://www.un.org/en/ga/search/view_doc.asp?symbol=A/RES/73/2. Accessed 13 Feb 2019.

[15] Rideout C, Gil R, Browne R (2013). Using the Delphi and snow card techniques to build consensus among diverse community and academic stakeholders. Prog Community Health Partnersh.

[16] Upadhyay RP (2012). An overview of the burden of non-communicable diseases in India. Iran J Public Health.

